# Ultra-low Doping on Two-Dimensional Transition Metal Dichalcogenides using DNA Nanostructure Doped by a Combination of Lanthanide and Metal Ions

**DOI:** 10.1038/srep20333

**Published:** 2016-02-03

**Authors:** Dong-Ho Kang, Sreekantha Reddy Dugasani, Hyung-Youl Park, Jaewoo Shim, Bramaramba Gnapareddy, Jaeho Jeon, Sungjoo Lee, Yonghan Roh, Sung Ha Park, Jin-Hong Park

**Affiliations:** 1 School of Electronics and Electrical Engineering, Sungkyunkwan University, Suwon 440-746, Korea; 2 Department of Physics, Sungkyunkwan University, Suwon 440-746, Korea; 3 SKKU Advanced Institute of Nanotechnology (SAINT), Sungkyunkwan University, Suwon 440-746, Korea

## Abstract

Here, we propose a novel DNA-based doping method on MoS_2_ and WSe_2_ films, which enables ultra-low n- and p-doping control and allows for proper adjustments in device performance. This is achieved by selecting and/or combining different types of divalent metal and trivalent lanthanide (Ln) ions on DNA nanostructures, using the newly proposed concept of Co-DNA (DNA functionalized by both divalent metal and trivalent Ln ions). The available n-doping range on the MoS_2_ by Ln-DNA is between 6 × 10^9^ and 2.6 × 10^10 ^cm^−2^. The p-doping change on WSe_2_ by Ln-DNA is adjusted between −1.0 × 10^10^ and −2.4 × 10^10 ^cm^−2^. In Eu^3+^ or Gd^3+^-Co-DNA doping, a light p-doping is observed on MoS_2_ and WSe_2_ (~10^10 ^cm^−2^). However, in the devices doped by Tb^3+^ or Er^3+^-Co-DNA, a light n-doping (~10^10 ^cm^−2^) occurs. A significant increase in on-current is also observed on the MoS_2_ and WSe_2_ devices, which are, respectively, doped by Tb^3+^- and Gd^3+^-Co-DNA, due to the reduction of effective barrier heights by the doping. In terms of optoelectronic device performance, the Tb^3+^ or Er^3+^-Co-DNA (n-doping) and the Eu^3+^ or Gd^3+^-Co-DNA (p-doping) improve the MoS_2_ and WSe_2_ photodetectors, respectively. We also show an excellent absorbing property by Tb^3+^ ions on the TMD photodetectors.

Deoxyribonucleic acid (DNA) nanostructures are currently being considered one of the promising materials for next-generation nanotechnology owing to the self-assembly and highly selective binding properties of DNA, which are based on the Watson-Crick complementary rules^1^,^2^,^3^,^4^,^5^. The self-assembled DNA nanostructures have been recently used in various nanoscale research fields, such as spintronics^6^,^7^, nanoelectronics^8^,^9^, biosensors^10^,^11^, and nanophotonics^12^,^13^. In particular, due to the highly selective binding property of DNA nanostructures, various particles such as metal nanoparticles (NPs)^14^,^15^, protein molecules^16^, nanowires (NWs)^17^, and divalent metal ions^18^,^19^,^20^,^21^ have been successfully incorporated onto the bases and backbone sites of DNA, eventually functionalizing the DNA nanostructures. Braun *et al.*^22^ and Maune *et al.*^23^ also reported self-assembled single-walled carbon nanotubes (SWNTs) bound on natural and origami DNA templates.

Meanwhile, transition metal dichalcogenides (TMDs) with a two-dimensional layered structure, such as molybdenum disulfide (MoS_2_) and tungsten diselenide (WSe_2_), have been considered highly promising materials for next-generation flexible, wearable, stretchable and transparent devices due to their unique physical, electrical and optical properties^24^,^25^,^26^,^27^,^28^,^29^,^30^. TMD-based electronic devices are immune to short-channel effects owing to their excellent thickness scalability down to an atomic monolayer and the van der Waals epitaxial structure without dangling bonds^24^,^25^. Moreover, TMD materials are also expected to be suitable in optoelectronic applications because of their tunable energy bandgap property which is controllable by layer thickness (from 1.8 eV to 1.2 eV) and noble quantum efficiency^28^,^29^,^30^. In particular, recent studies on TMD devices have focused on developing a suitable doping technique because precise control of the threshold voltage (V_TH_) and the number of tightly-bound trions^31^ as well as very low contact resistance are required to achieve high performance. It is especially critical to develop an ultra-low level doping technique for the proper design and optimization of TMD-based devices (V_TH_ for transistors and tightly-bound trions for photodetectors) because high level doping (about 10^12^ cm^−2^) causes TMD to act as a near-metallic layer. However, it is difficult to apply an ion implantation technique to TMD materials to achieve low-level doping due to crystal damage that occurs during the implantation process. Although safe doping techniques have recently been developed which can be applied to TMDs with an atomic layer structure, most of the previous TMD doping techniques employing potassium^32^, Au NPs^33^, polyethyleneimine (PEI)^34^, functional self-assembled monolayers (SAMs)^35^, and plasma treatment with fluorine (F) or oxygen (O)^36^ presented very high doping levels of ~10^12 ^cm^−2^. Recently, low-level n- and p-doping of TMD materials was achieved using cesium carbonate (Cs_2_CO_3_)^37^, octadecyltrichlorosilane (OTS)^38^ and M-DNA^39^, but further studies are needed to reduce the doping level down to an intrinsic level.

Here, we demonstrate a novel DNA-based doping method on selected 2D TMD materials (MoS_2_ and WSe_2_, which represent n- and p-channel materials, respectively), enabling ultra-low-level n- and p-doping control, which had not been previously accomplished. This is achieved by selecting and/or combining different types of positive ions on DNA nanostructures. The DNA is functionalized by trivalent lanthanide ions (Ln-DNA) or both divalent metal and trivalent lanthanide ions (Co-DNA), which is newly proposed in this work. Since the phosphate backbone (PO_4_^−^) in DNA causes n-doping on the TMD films by attracting and holding hole carriers on the side of TMD, various selections and/or combinations of divalent and trivalent positive ions on DNA make it possible to achieve both very low-level n- and p-doping, which are very close to the intrinsic level. First of all, Ln-DNA nanostructures based on double-crossover (DX) DNA lattices are explored as a function of ion concentration (here, Gd^3+^) through Raman spectroscopy and atomic force microscopy (AFM) analyses. We then investigate low-level n- and p-doping phenomena on MoS_2_ and WSe_2_ by Ln-DNA and Co-DNA in terms of the performance (threshold voltage, on-/off-currents, photoresponsivity, and detectivity) of electronic and optoelectronic devices using Raman spectroscopy, photoluminescence (PL) spectroscopy, and electrical/optical measurements (*I*_D_ − *V*_G_ with/without exposure to 655-nm, 785-nm, and 850-nm lasers). We also discuss the absorption of trivalent lanthanide (Ln) ions with/without DNA templates with respect to the specific wavelengths of light and its influence on the performance of Ln-DNA or Co-DNA-doped TMD optoelectronic devices.

## Results and Discussion

### Synthesis and analysis of Ln-DNA nanostructures

**Figure 1:**
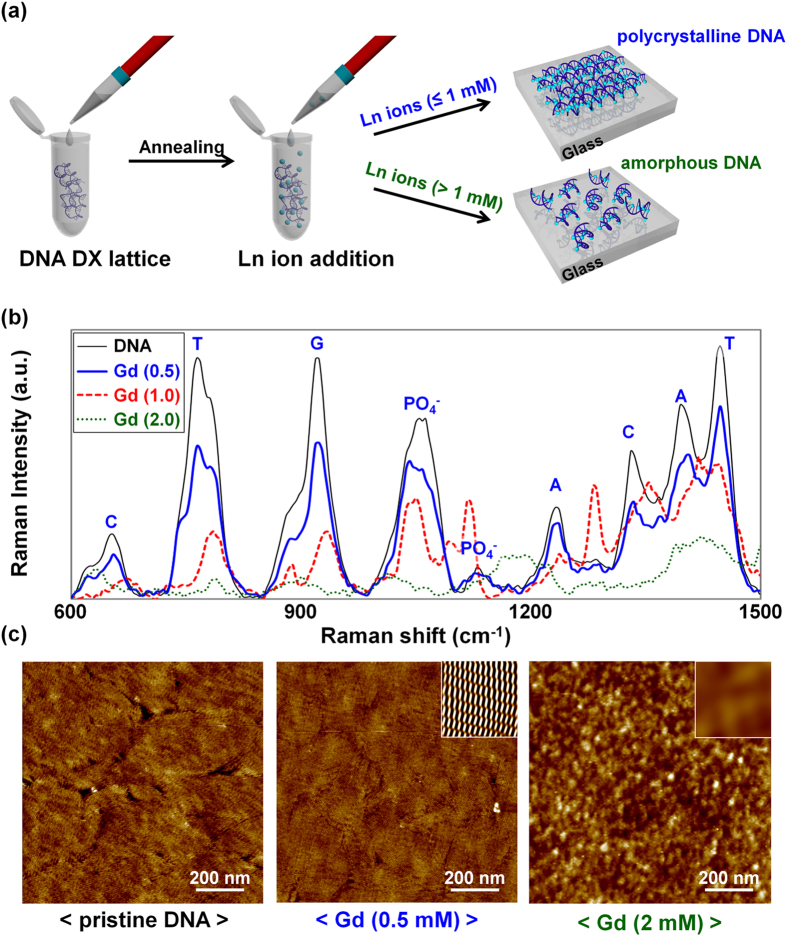
Schematic diagrams showing the Ln-DNA DX lattice fabrication process and the Raman spectra and AFM images of Ln-DNA with various concentrations of lanthanide ions. (**a**) Schematic diagrams of Ln-DNA DX lattice fabrication. (**b**) Raman spectra of DNA and Gd-DNA with 0.5, 1.0 and 2.0 mM of Gd^3+^ ions. (**c**) AFM images of pristine DNA and Gd-DNA with 0.5 and 2.0 mM Gd^3+^. Inset: Noise-filtered 2D spectrum images constructed by Fast Fourier Transform showing a crystalline nature with clear periodicity of DNA lattices with 0.5 mM of Gd^3+^ and an amorphous nature without periodicity of DNA with 2.0 mM of Gd^3+^.

As shown in Fig. 1a, we formed a DNA double-crossover (DX) lattice structure with various concentrations of lanthanide ions and then investigated its stability by Raman spectroscopy and AFM to avoid structural deformation of the DX lattices. The DNA DX lattices were fabricated by a conventional solution-free annealing process. After annealing at 95 °C, various concentrations of Ln ions were added into the DX lattice solution (0, 0.5, 1 and 2 mM of Eu^3+^, Gd^3+^, Tb^3+^, and Er^3+^). In order to verify the stability of Gd-DNA, which is Ln-DNA with various concentrations of Gd^3+^, we obtained Raman spectra of Gd-DNA grown on glass substrate (Fig. 1b) and identified peak differences between 0 mM (pristine DNA), 0.5 mM, 1 mM, and 2 mM of [Gd^3+^]. In the pristine DNA crystal without Gd^3+^, Raman peak signals were observed at 1246 and 1420 cm^−1^ for adenine (A), 770 and 1469 cm^−1^ for thymine (T), 931 and 1590 cm^−1^ for guanine (G), 618, 655, and 1348 cm^−1^ for cytosine (C), and 1066 and 1145 cm^−1^ for the phosphate backbone (PO_4_^−^)^40^,^41^. After binding Gd^3+^ ions onto the base pairing and backbone sites, the Raman intensity related to base and backbone sites was reduced. In particular, a Raman peak shift was observed in the base pairing sites only when the concentration of [Gd^3+^] was above 0.5 mM. Because the Ln ions (including Gd^3+^) were reported to be normally attached to backbone sites due to its ionic radius (300 ~ 340 pm), which is larger than the hydrogen bonding length (340 pm) in DNA bases^42^, it is thought that the Ln ions are intercalated in the bases without chemical bonding, and therefore seem to influence the DNA nanostructure. As the concentration of [Gd^3+^] increased, the Raman peak intensity of Gd-DNA decreased, and it was eventually hard to identify Raman peaks of Gd-DNA above 1 mM of [Gd^3+^], indicating that the Gd-DNA crystal structure is deformed when more than 1 mM [Gd^3+^] ions are added. This was verified once again through AFM images of Gd-DNA with 0 mM (pristine DNA), 0.5 mM, and 2 mM of [Gd^3+^] in Fig. 1c. The AFM images of pristine and Gd (0.5 mM) samples showed polycrystalline structures without deformation, and the clear periodicity of each DX lattice was also confirmed in the noise-filtered 2D spectrum image after fast Fourier transform (FFT). However, in the case of 2 mM [Gd^3+^], Gd-DNA complexes seemed to be aggregated, and consequently form an amorphous structure. A similar phenomenon was also observed in the cases of other Ln ions with different concentrations, and the determined optimum concentrations of each Ln ion were 1 mM for [Eu^3+^], 1 mM for [Gd^3+^], 1 mM for [Tb^3+^], and 1 mM for [Er^3+^].

### Raman analysis of TMD films doped by Ln-DNA or Co-DNA

**Figure 2:**
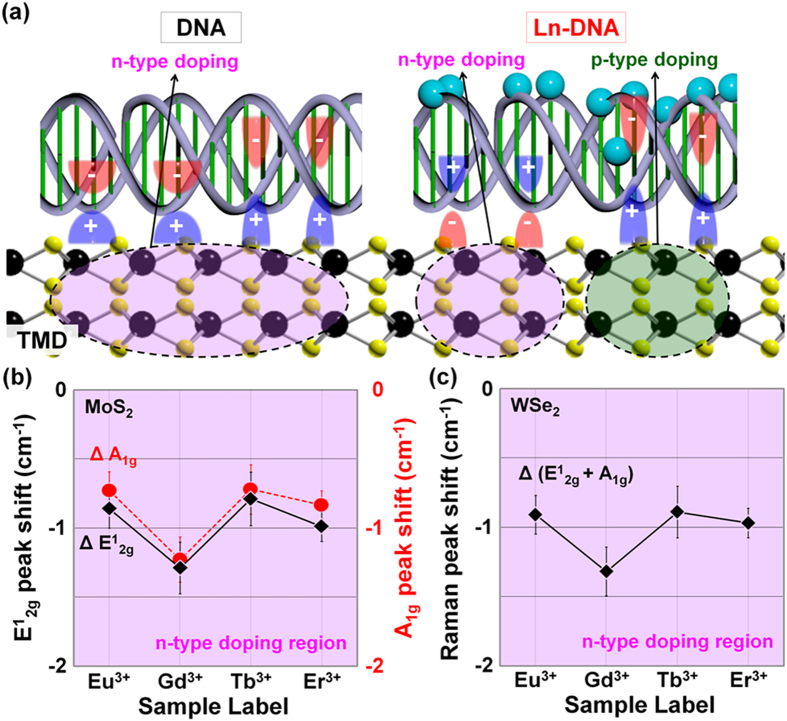
Doping mechanism and Raman analysis of TMD films doped by DNA or Ln-DNA. (**a**) Schematic diagrams. Raman peak shift values of the (**b**) MoS_2_ and (**c**) WSe_2_ doped by Ln-DNA.

The artificially designed Ln- or Co-DNA DX solution was dropped and dried five times on the MoS_2_ and WSe_2_ surfaces. DNA nanostructures were homogeneously dispersed along the surface of MoS_2_ and WSe_2_ because of the self-aligning capability of the DNA^39^. In the previous study, the PO_4_^−^ backbone sites in DNA nanostructures were reported to induce and hold positive charges (holes) at the interface region in the side of the TMD, resulting in n-doping (Fig. 2a). In the case of Ln-DNA, Ln ions are predominantly attached in the DNA backbone sites due to their relatively larger ionic radii so that the attached Ln ions are expected to compete with the negative charges of base pairings, leading to relatively light n- or p-type doping on TMD films. This would be expected because previous research showed a reduction in the p-type doping level of M-DNA by using a metal ion with a relatively larger ionic radius^39^. Raman analysis was then performed on the Ln-DNA-doped TMD samples to investigate the Ln-DNA doping effects by Eu^3+^, Gd^3+^, Tb^3+^, and Er^3+^ ions on TMD films, as shown in Supplementary Information Fig. S3. In the MoS_2_ films, two conventional peaks (E^1^_2g_ and A_1g_) were observed at ~380 cm^−1^ and ~406 cm^−1^, which respectively indicate the in-plane and out-of-plane vibrations for bulk MoS_2_^43^. On the other hand, only a single peak was obtained at ~250 cm^−1^ in WSe_2_ films because both the E^1^_2g_ and the A_1g_ modes for WSe_2_ are close to 250 cm^−1^ 44. To clarify the degree of Ln-DNA doping by each ion on TMD films, we extracted the peak shift values in E^1^_2g_, A_1g_, and E^1^_2g_ + A_1g_ before and after Ln-DNA doping, and we plotted the values in Fig. 2b,c. Here, we prepared 10 different samples in each doping condition for Raman analysis and measured at five different points in each sample. As shown in Fig. 2b,c, a red-shift phenomenon occurred in the E^1^_2g_, A_1g_, and E^1^_2g_ + A_1g_ peaks which have respectively peak shift values in the ranges of −1.3 ~ −0.8 cm^−1^, −1.2 ~ −0.7 cm^−1^, and −1.3 ~ −0.9 cm^−1^, indicating that very low level n-type doping was achieved on the TMD films. This n-type doping phenomenon by Ln-DNA also seems to be slightly weaker than that by pristine DNA. For reference, a blue-shift phenomenon in all of the peaks was observed after the M-DNA doping on the MoS_2_ and WSe_2_ films (2.8 ~ 5.0 cm^−1^ for E^1^_2g_, 3.8 ~ 5.2 cm^−1^ for A_1g_, and 3.1 ~ 5.1 cm^−1^ for E^1^_2g_ + A_1g_)^39^, indicative of p-type doping.

**Figure 3:**
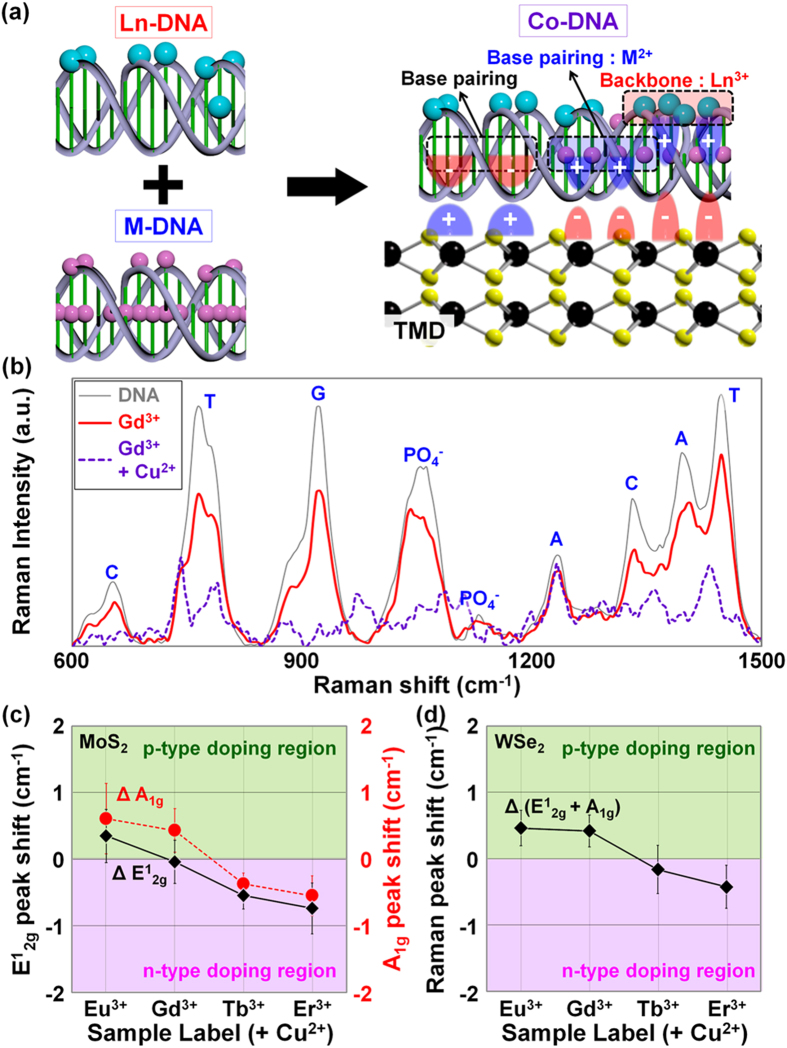
Doping mechanism by Co-DNA and Raman analysis of TMD films doped by Co-DNA. (**a**) Schematic diagrams explaining Co-DNA doping on TMD. (**b**) Raman spectra of DNA, Gd^3+^-based Ln-DNA (or Gd-DNA), and Gd^3+^-based Co-DNA (with Cu^2+^ ions). (**c**) Extracted Raman peak shift data of MoS_2_ and WSe_2_ doped by Co-DNA.

Next, we added Cu^2+^ ions with a relatively smaller ionic radius into Ln-DNA to acquire weaker n- or p-type doping effects on TMD films by converting the negative charges at the base pairings to positive charges; these new DNA nanostructures are referred to as Co-DNA. Here, the Cu^2+^ ions are expected to be bound at DNA base pairings because the Ln ions are predominantly attached to the backbone sites, eventually modulating the strength of the total charge which previously showed weak negative polarity. In this Co-DNA experiment, the different amounts of Ln ions (0.5 mM Eu^3+^, 0.5 mM Gd^3+^, 0.5 mM Tb^3+^, or 0.5 mM Er^3+^) were mixed with 2 mM of Cu^2+^ ions to avoid structural deformation of Co-DNA nanostructures. Then, Raman spectroscopy was performed on the DNA (pristine), Ln-DNA (by Gd^3+^), and Co-DNA (by Gd^3+^ and Cu^2+^) structures, as shown in Fig. 3b. As previously mentioned, the intensity of Raman peaks, indicating the base pairing and backbone sites of DNA, was reduced in the case of Gd-DNA due to the Gd^3+^ ions attached on the sites. When Cu^2+^ ions were added into the Gd-DNA, we observed an additional reduction in all of the peaks related to base pairings and backbone sites, indicating that Cu^2+^ ions were additionally attached on the remaining backbone sites as well as the base pairings; it was previously predicted that Ln ions were not bound to the base pairings. Finally, we coated the various Co-DNA nanostructures on MoS_2_ and WSe_2_ films, and performed Raman analysis on the films. The raw Raman peak data of Co-DNA-doped MoS_2_ and WSe_2_ can be found in Supplementary Information Fig. S3. Figure 3c,d show the extracted peak shift values before/after Co-DNA doping for MoS_2_ (ΔE^1^_2g_ and ΔA_1g_) and WSe_2_ (ΔE^1^_2g_ + A_1g_), respectively. Here, the Raman measurement was performed on fifty different points in each sample. For both the MoS_2_ and WSe_2_ cases, Co-DNA nanostructures with Tb^3+^ or Er^3+^ ions seem to induce much weaker n-type doping phenomena compared to the previous Tb- and Er-DNA (Ln-DNA). This is because smaller peak shift values were observed in the peaks (−0.5 ~ −0.7 cm^−1^ for E^1^_2g_, −0.3 ~ −0.5 cm^−1^ for A_1g_, and −0.2 ~ −0.4 cm^−1^ for E^1^_2g_ + A_1g_). Meanwhile, a blue-shift phenomenon was confirmed in the MoS_2_ and WSe_2_ films doped by Co-DNA with Eu^3+^ or Gd^3+^ ions, indicating that the films were lightly p-doped. Here, the peak shift values are 0.3 ~ 0.1 cm^−1^ for E^1^_2g_, 0.6 ~ 0.4 cm^−1^ for A_1g_, and 0.45 ~ 0.4 cm^−1^ for E^1^_2g_ + A_1g_, which are much smaller than the previously reported values of M-DNA-doped flims^24^. These results support the claim that the Cu^2+^ ions are bound at DNA base pairings (also, remained backbone sites) and can be used to modulate the strength of total charge in Ln-DNA, eventually making the Co-DNA nanostructure show weak negative or positive polarity. In particular, based on the ionic radius and optimum concentrations shown in Table 1, it is thought that Eu^3+^ ions show the strongest positive charge due to their largest ionic radius and highest concentration. As expected, the largest peak shift was observed in the MoS_2_ and WSe_2_ films doped by Co-DNA with Eu^3+^ ions. In contrast to the Eu^3+^ sample, the smallest peak shift, indicating light n-type doping, was obtained in the TMD films doped by Co-DNA with Er^3+^ ions which are expected to show the lowest positive charge strength owing to the smallest ionic radius.

### Electrical characteristics of Ln-DNA- and Co-DNA-doped TMD electronic devices

**Figure 4:**
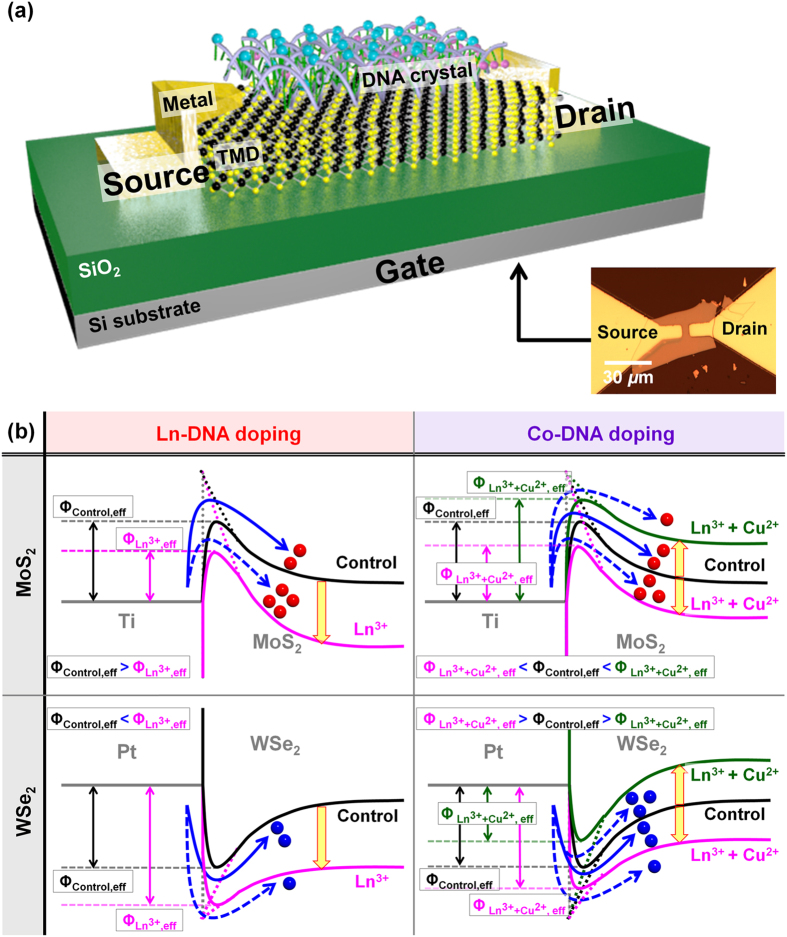
Schematic diagram of back-gated TMD doped device and Ln- or Co-DNA doping effect on TMD films. (**a**) Schematic diagram showing the back-gated transistor (or photodetector) device fabricated on TMD films doped by Ln- or Co-DNA. (**b**) Energy band diagrams of source-undoped/doped TMD junctions.

We then fabricated TMD electronic devices (back-gate transistors) and investigated the electrical properties (threshold voltage, 2D sheet carrier concentration, and on-current) of TMD devices before/after Ln-DNA or Co-DNA doping. Figure 4 shows the schematic diagram of TMD transistors and the energy band diagrams of metal-undoped/doped TMD junctions in the source-side when proper operating biases (positive *V*_DS_ for MoS_2_ and negative *V*_DS_ for WSe_2_) are applied. When Ln-DNA is coated on TMD films, as already explained, the negative charges of Ln-DNA are expected to hold hole carriers at the interface between Ln-DNA and TMD, resulting in an n-type doping phenomenon. In the case of MoS_2_, as shown in Fig. 4b, the electric field at the source-side Ti-MoS_2_ junction is predicted to increase due to the down-shift of MoS_2_ energy bands by Ln-DNA doping, thereby reducing the effective barrier height of the Ti-MoS_2_ junction (Schottky barrier lowering effect: *Ф*_Control,eff_ > *Ф*_Ln_^3+^_,eff_). As a result, more electron carriers may be injected from Ti to MoS_2_, and this result in a negative shift in *V*_TH_. In addition, it is also predicted that the WSe_2_ energy band shifts down after Ln-DNA doping. Subsequently, the effective hole barrier height from the source to WSe_2_ increases, finally causing a negative shift in *V*_TH_. On the other hand, because Co-DNA shows very weak positive polarity (Eu^3+^+Cu^2+^ or Gd^3+^+Cu^2+^) or negative polarity (Tb^3+^+Cu^2+^ or Er^3+^+Cu^2+^), it is possible to hold electron or hole carriers at the interface, respectively, eventually causing very low level n- or p-type doping. In particular, the level of n-doping by Tb- or Er-based Co-DNA is expected to be lower than that by Ln-DNA. In MoS_2_, Eu- or Gd-based Co-DNA moves up the MoS_2_ energy band, consequently reducing the electric field at the source-side junction and increasing the effective electron barrier height (green line; a positive shift in *V*_TH_). In contrast, Tb- or Er-based Co-DNA causes n-doping on MoS_2_, thereby shifting down its energy band and eventually decreasing the effective electron barrier height (pink line; a negative shift in *V*_TH_). A similar phenomenon in the effective hole barrier height and *V*_TH_ is also expected on WSe_2_, as shown in the right-bottom of Fig. 4b.

**Figure 5:**
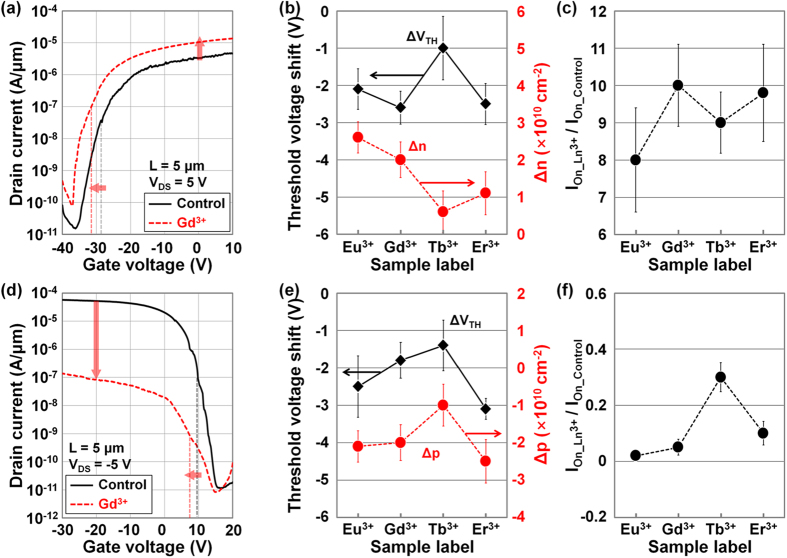
Electrical characterization of TMD transistors doped by Ln-DNA. *I*_D_ − *V*_G_ characteristics of the (**a**) MoS_2_ and (**d**) WSe_2_ transistors undoped/doped by Gd^3+^-DNA. Threshold voltage shifts (Δ*V*_TH_ = *V*_TH_Ln-DNA_ – *V*_TH_Control_) and variations of carrier concentration (Δ*n* = *n*__Ln-DNA_ – *n*__Control_ and Δ*p* = *p*__Ln-DNA_ – *p*__Control_) extracted in (**b**) MoS_2_ and (**e**) WSe_2_ transistors, which were undoped/doped by Ln-DNA. On-current ratio (*I*_on_ ratio = *I*_on_Ln-DNA_/*I*_on_Control_) extracted in the undoped/doped (**c**) MoS_2_ and (**f**) WSe_2_ transistors.

Figure 5a shows *I*_D_ − *V*_G_ characteristics of undoped and Gd-DNA-doped MoS_2_, which were measured at *V*_DS_ = 5 V. As previously predicted, a negative shift in *V*_TH_ (from −28.5 V to −31.2 V) and an increase in on-current (from 2.3 × 10^−6 ^A/μm to 1.2 × 10^−5 ^A/μm) were observed after the Gd-DNA doping, indicating that the Gd-DNA caused n-type doping of MoS_2_. These changes are attributed to an increase in the tunneling probability from the source to the MoS_2_ channel by the Gd-DNA doping. These changes in *V*_TH_ and on-current were also observed in the other Ln-DNA with Eu^3+^, Tb^3+^, or Er^3+^ ions, as shown in Fig. 5b,c. The extracted ∆*V*_TH_ (=*V*_TH_Ln-DNA_ − *V*_TH_Control_) values were between −1 and −2.5 V, indicating the n-doping of MoS_2_, which is also consistent with the previous Raman analysis. The difference in 2D sheet doping concentrations (∆*n* = *n*_Ln-DNA_−*n*_Control_) extracted in the Ln-DNA-doped MoS_2_ devices also showed positive values, which mean an increase in the number of electron carriers and consequently in n-doping of MoS_2_. The lowest ∆*n* was ~6 × 10^9 ^cm^−2^ in the case of Tb-DNA, and the available n-doping range by Ln-DNA was between ~6 × 10^9 ^cm^−2^ and ~2.6 × 10^10 ^cm^−2^, in which the device performance can be controlled. In addition, we confirmed the improvements in on-current through Ln-DNA doping in Fig. 5c. The on-current ratio (=*I*_On_Ln-DNA_/*I*_On_Control_) was between approximately 8 and 10. This enhancement is thought to be caused by the reduction in effective electron barrier height through Ln-DNA doping, indicating that there was a reduction in contact resistance. Based on simple on-state contact resistance extraction^45^, Gd-DNA doping reduced the estimated contact resistance to ~72.2 kΩ (from ~90.7 kΩ) on the MoS_2_ device and increased it to 95.1 kΩ (from ~67.7 kΩ) on the WSe_2_ device. On the other hand, we observed a degradation in the electronic performance of WSe_2_ devices after Ln-DNA doping, as shown in Fig. 5d, which presents the *I*_D_ − *V*_G_ characteristics of undoped/doped WSe_2_ devices by Gd-DNA (at *V*_DS_ = −5 V). Here, a negative shift in *V*_TH_ (from 10 V to 8 V) and a decrease in on-current (from 5.2 × 10^−5 ^A/μm to 9.2 × 10^−8 ^A/μm) were confirmed, indicating the n-doping of WSe_2_. Then, ∆*V*_TH_ values extracted in each Ln-DNA-doped WSe_2_ device were between −1.4 V and −3.1 V; we also obtained negative ∆*p* values (=*p*_Ln-DNA_−*p*_Control_) in the range from −1.0 × 10^10 ^cm^−2^ to −2.4 × 10^10 ^cm^−2^ (Fig. 5e). These changes are consistent with the red-shift phenomenon in the Raman peaks mentioned above, which is indicative of n-doping (reduction of hole concentration in WSe_2_). In contrast to the improvement of on-current in MoS_2_ devices, Ln-DNA doping degraded the parameters of WSe_2_ devices, as shown in Fig. 5f. The on-current ratio was found to be below 0.3, likely because the energy band of WSe_2_ was shifted down after Ln-DNA doping. Consequently, its effective hole barrier height from the source to WSe_2_ was increased, resulting in an increase in contact resistance.

**Figure 6:**
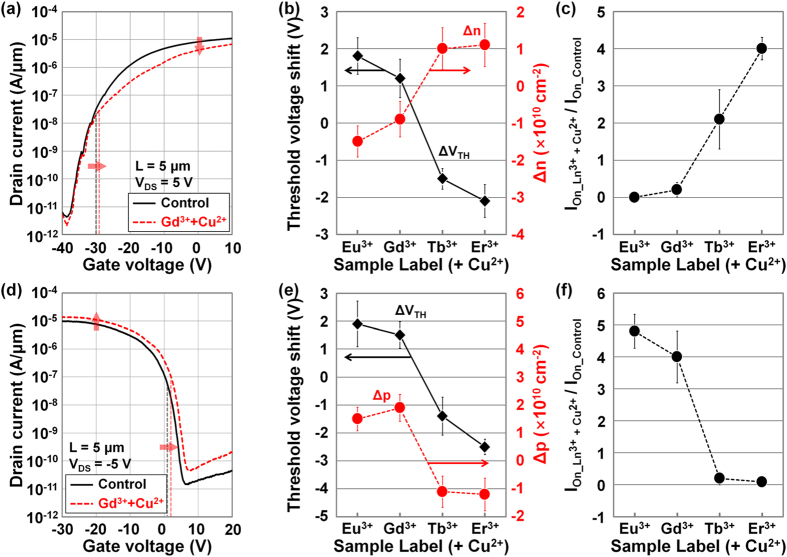
Electrical characterization of TMD transistors doped by Co-DNA. *I*_D_–*V*_G_ characteristics of the (**a**) MoS_2_ and (**d**) WSe_2_ transistors undoped/doped by Gd^3+^-based Co-DNA. Threshold voltage shifts (Δ*V*_TH_ = *V*_TH_Ln-DNA_ – *V*_TH_Control_) and variations of carrier concentration (Δ*n* = *n*__Ln-DNA_ – *n*__Control_ and Δ*p* = *p*__Ln-DNA_ – *p*__Control_) extracted in (**b**) MoS_2_ and (**e**) WSe_2_ transistors, which were undoped/doped by Co-DNA. On-current ratio (*I*_on_ ratio = *I*_on_Ln-DNA_/*I*_on_Control_) extracted in the undoped/doped (**c**) MoS_2_ and (**f**) WSe_2_ transistors.

However, as already expected, slightly different changes were observed on the TMD-based electronic devices after doping by the Co-DNA. Figure 6a shows the *I*_D_−*V*_G_ characteristics of MoS_2_ devices undoped and doped by Co-DNA (Gd^3+^+Cu^2+^), where a positive shift in *V*_TH_ (from –30 V to –28.2 V) and a reduction in on-current (from 8.2 × 10^−5 ^A/μm to 4.2 × 10^−5 ^A/μm) were observed after the Gd^3+^-based Co-DNA doping, indicating a light p-doping of MoS_2_. Based on the extracted ∆*V*_TH_ (=*V*_TH_Co-DNA_ − *V*_TH_Control_) and ∆*n* (=*n*_Co-DNA_−*n*_Control_) values of MoS_2_ devices doped by various Co-DNA in Fig. 6b, Co-DNA seems to cause a light p-doping phenomenon on MoS_2_ where Eu^3+^ or Gd^3+^ ions were incorporated. Here, we observed positive ∆*V*_TH_ values above 1.2 V and negative ∆*n* values below −9 × 10^9 ^cm^−2^. This p-doping concentration is slightly lower than those of the other TMD doping techniques using OTS (~1.6 × 10^10^ cm^−2^)^38^ or M-DNA (~2.3 × 10^10^ cm^−2^)^39^. Compared to the Eu- or Gd-DNA (Ln-DNA), the additionally inserted Cu^2+^ ions are expected to be successfully combined with the DNA base pairings and remaining backbone sites. This consequently provides very light p-doping of MoS_2_ because the strength of negative charges is reduced and the effective electron barrier height is increased by the up-shift of the MoS_2_ energy band. However, a light n-doping phenomenon (negative ∆*V*_TH_ below −1.5 V and positive ∆*n* above 10^10^ cm^−2^) was interestingly observed in the MoS_2_ devices doped by Co-DNA with Tb^3+^ or Er^3+^ ions. This n-doping concentration is also lower than those of the other doping techniques using Cs_2_CO_3_ (~1 × 10^11 ^cm^−2^)^37^ or pristine DNA (~6.4 × 10^10 ^cm^−2^)^39^. Compared to the cases of Eu- or Gd-DNA (Ln-DNA), fewer Cu^2+^ ions are attached to the Tb- and Er-DNA, eventually allowing the polarity of the Co-DNA to remain negative. We also note that all of these changes in the MoS_2_ device performance coincide with the previously mentioned Raman peak shifting behavior for the various kinds of Ln ions. This difference (between n- and p-doping by Co-DNA) was clearly observed in the on-current ratio, which was plotted in Fig. 6c. As expected for the MoS_2_ devices p-doped by Co-DNA with Eu^3+^ or Gd^3+^ ions, the on-currents were reduced 0.06 ~ 0.2 times compared to the initial values of the control samples. However, in the cases where Tb^3+^ and Er^3+^ ions were used (n-doping), a 2.1 ~ 4-fold increase in the on-current was observed after Co-DNA doping.

A similar doping phenomenon by Co-DNA was observed on WSe_2_ films, as shown in Fig. 6d. The Gd^3+^-based Co-DNA caused p-doping on WSe_2_ as well as on MoS_2_ so that a positive shift in V_TH_ and an increase in on-current were observed due to the up-shift in the WSe_2_ energy band by p-doping and the consequent reduction of the effective hole barrier height at the source-side Pt-WSe_2_ junction. Figure 6e shows the ∆*V*_TH_ and ∆*p* extracted in the WSe_2_ devices doped by various Co-DNA, which also presents the difference between p- and n-doping effects. In the case of Eu^3+^- and Gd^3+^-based Co-DNA doping, we obtained positive ∆*V*_TH_ (1.5 ~ 1.9 V) and ∆p (1.4 × 10^10^ ~ 1.9 × 10^10 ^cm^−2^) values, which indicate p-doping of WSe_2_. The other Tb^3+^- and Er^3+^-based Co-DNA resulted in negative ∆*V*_TH_ and ∆*p* values (n-doping of WSe_2_), which were, respectively, below −1.4 V and −1.1 × 10^10 ^cm^−2^. As seen in Fig. 6f, the on-current ratio of the WSe_2_ devices before/after doping was also increased up to 4.1 when Eu^3+^- and Gd^3+^-based Co-DNA doping (p-doping) was performed. However, the on-current ratio was reduced below 0.25 in devices doped with Tb^3+^- and Er^3+^-based Co-DNA (n-doping). In additional experiments on tri-layer TMDs, we confirmed that the doping effects of Ln- or Co-DNA were independent of TMD thickness. We also note that the influence of four kinds of solvents used during the fabrication process (DI water, acetone, IPA, and buffer solutions) on TMD-based devices (here, ΔV_TH_) seems to be negligible when compared to the effect of Ln- or Co-DNA-doping (0.3–0.33 V for DI water, 0.2–0.22 V for acetone, −0.24–−0.2 V for IPA, and 0.17–0.23 V for the buffer solution). In addition, the *V*_TH_ values of the Ln- and Co-DNA-doped MoS_2_ and WSe_2_ devices changed slightly after 120 hours of air-exposure (Supplementary information Fig. S7). The ∆*V*_TH_ values of Ln-DNA-doped MoS_2_ and WSe_2_ transistors increased 15–20%, indicating the weakening of the n-type doping effects after 120 hours. The ∆*V*_TH_ values of the Co-DNA-doped samples also decreased (Eu- or Gd-based Co-DNA) or increased (Tb- or Er-based Co-DNA) as a function of air exposure time because of the reduced dipole moment of the phosphate backbone (PO_4_^−^) and lanthanide ions (Ln^3+^) related to the humidity-associated structural deformation of Ln- and Co-DNA nanostructures^46^.

### Characteristics of Co-DNA-doped TMD optoelectronic devices

**Figure 7:**
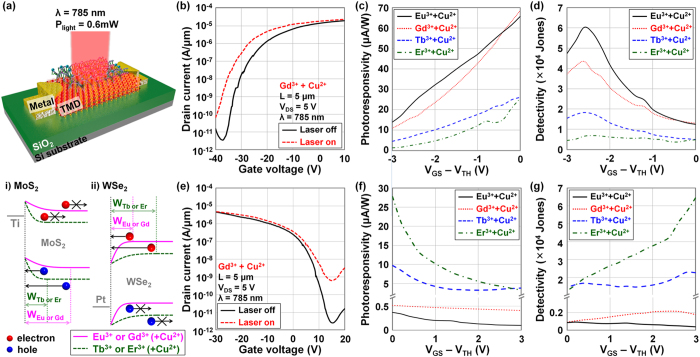
Schematic diagram and characterization of TMD photodetectors doped by various Co-DNA. (**a**) Schematic diagram of Co-DNA-doped MoS_2_/WSe_2_ photodetectors with a light source (*λ* = 785 nm and *P* = 0.6 mW), and the energy band diagrams of Ti-MoS_2_/Pt-WSe_2_ junctions under the illuminated condition. *I*_D_-*V*_G_ characteristics of Gd^3+^-based Co-DNA-doped (**b**) MoS_2_ and (**e**) WSe_2_ photodetectors before/after the laser exposure. Photoresponsivity of (**c**) MoS_2_ and (**f**) WSe_2_ photodetectors as a function of *V*_GS_–*V*_TH_ (in off-state). Detectivity of (**d**) MoS_2_ and (**g**) WSe_2_ photodetectors as a function of *V*_GS_–*V*_TH_ (in off-state).

We then performed a photocurrent measurement on the Co-DNA-doped TMD photodetector devices with 655-nm, 785-nm, and 850-nm lasers in order to investigate the effects of p- and n-doping by Co-DNA on optoelectronic device performance. Figure 7a shows a schematic diagram of the Co-DNA-doped TMD photodetector, along with the corresponding energy band diagrams of i) MoS_2_ and ii) WSe_2_ before/after Co-DNA doping. Compared to the control device, the depletion width at the source/MoS_2_ junction is expected to be broadened after Eu^3+^ or Gd^3+^-based Co-DNA doping (pink solid line: p-doping), consequently helping to collect photocarriers and increase photocurrent. However, the n-doping phenomenon by Tb^3+^ or Er^3+^-based Co-DNA is predicted to narrow the depletion width and reduce photocurrent below that of the control device (green dotted line: n-doping). In contrast, WSe_2_ photodetectors doped by the Tb^3+^ or Er^3+^-based Co-DNA show higher photocarrier collection and subsequently higher photocurrent. Figure 7b shows *I*_D_ − *V*_G_ characteristics of a Gd^3+^-based Co-DNA-doped TMD photodetector before and after light exposure. In the off-state (*V*_GS_ < *V*_TH_), the absence of majority carrier paths in MoS_2_ (low electron current) and the high hole barrier height (low hole current) are expected to reduce the dark current below a level of 10^−11 ^A/μm. As a result, the photocurrent was more clearly observed in the off-state because of the low dark current level. We then extracted and plotted photoresponsivity (*R* = *I*_Photo_/*P*_Light_) and detectivity (D* = (*RA*^1/2^)/(2*eI*_Dark_)^1/2^) values as a function of *V*_GS_ − *V*_TH_ in Fig. 7c,d. Here, the generated photocurrent is *I*_Laser_on_−*I*_Laser_off_, *P*_Light_ is the total incident optical power, A is the effective area of the device, *e* is the absolute value of the electron charge (1.6 × 10^−19 ^C), and I_Dark_ is dark current. In Fig. 7c, Eu^3+^ or Gd^3+^-based Co-DNA-doped MoS_2_ devices showed higher photoresponsivity (~68 μA/W at *V*_GS_ = *V*_TH_) than those (~25 μA/W at *V*_GS_ = *V*_TH_) of Tb^3+^ or Er^3+^-based Co-DNA samples because of their broader depletion width and subsequent higher photocurrent. The photoresponsivity was increased as a function of *V*_GS_ in all cases of Co-DNA because the conductivity of the electron channel is improved as *V*_GS_ approaches *V*_TH_. An opposite trend was observed in detectivity (D*) because D* is expressed as the ratio of *I*_Photo_ to *I*_Dark_ and is also affected by the *I*_Dark_, as shown in Fig. 7d. The highest value (maximum D* = ~6.02 × 10^4^ Jones at *V*_GS_ − *V*_TH_ = −2.6 V) was obtained in the Eu^3+^-based Co-DNA-doped device. Figure 7e shows *I*_D_ − *V*_G_ characteristics of the WSe_2_ device doped by Gd^3+^-based Co-DNA before/after light exposure, and we also extracted photoresponsivity and detectivity as a function of *V*_GS_ − *V*_TH_ in Fig. 7f,g. An opposite trend was observed in photoresponsivity of WSe_2_ devices compared to MoS_2_ devices; that is, higher values were obtained in the n-doped devices by Tb^3+^- and Er^3+^-based Co-DNA. In the Er^3+^-based Co-DNA-doped device, a maximum photoresponsivity of ~27 μA/W was observed at *V*_GS_ = *V*_TH_. In addition, like the MoS_2_ photodetectors, the photoresponsivity of WSe_2_ devices increased as the *V*_GS_ moved to the *V*_TH_ point. Detectivity was also better in the n-doped WSe_2_ devices, and it increased as a function of *V*_GS_ − *V*_TH_, where the maximum detectivity of ~6.5 × 10^4^ Jones was obtained in the Er^3+^-based Co-DNA-doped device when *V*_GS_ − *V*_TH_ = 3 V (~8.2 × 10^4^ Jones at *V*_GS_ − *V*_TH_ = 5.2 V).

**Figure 8:**
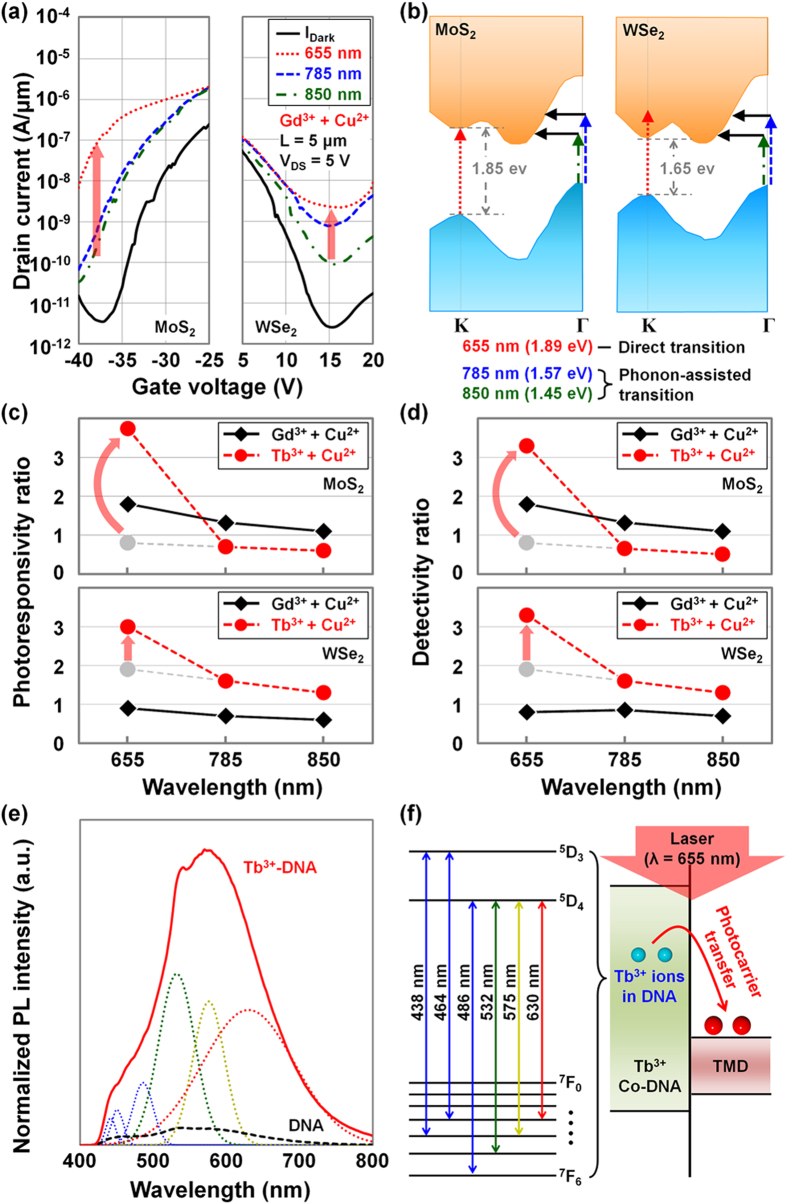
Characterization of Co-DNA-doped TMD photodetectors under laser exposure with different wavelengths. (**a**) *I*_D_−*V*_G_ characteristics of MoS_2_ and WSe_2_ photodetectors doped by Gd^3+^-based Co-DNA under laser exposure with different wavelengths (655, 785, and 850 nm). (**b**) Energy band structures of bulk MoS_2_ and WSe_2_. Here, we approximately marked the photon or phonon transition under the laser exposure with different wavelengths. (**c**) Photoresponsivity ratio (*R*_Co-DNA_/*R*_Control_) and (**d**) detectivity ratio (D*_Co-DNA_/D*_Control_) of Gd^3+^ and Tb^3+^-based Co-DNA-doped TMD photodetector under different laser exposures. (**e**) Photoluminescence data of DNA and Tb^3+^-DNA crystals, and Gaussian fitting lines for Tb^3+^-DNA PL data. Here, the excitation wavelength is 400 nm. (**f**) Energy state levels of Tb^3+^-DNA and the schematic presenting the mechanism of photocarrier transfer from Tb^3+^ ions to the TMD.

Then, the MoS_2_ and WSe_2_ photodetectors doped by Gd^3+^-based Co-DNA were investigated under laser exposure with different wavelengths (655-nm, 785-nm, and 850-nm), as shown in Fig. 8. Figure 8a shows *I*_D_ − *V*_G_ characteristics of the TMD photodetectors measured before and after the various light exposures. In both devices an increase in photocurrent was observed as the laser wavelength decreased. The energy of incident light increases with decreasing wavelength, and it eventually overcomes the direct bandgap (at K valley) at λ = ~655 nm (1.89 eV > 1.85 eV of direct E_g_MoS2_ and 1.65 eV of direct E_g_WSe2_), finally improving the absorption probability as seen in Fig. 8b. The photoresponsivity and detectivity ratio values (*R* = *R*_Co-DNA_/*R*_Control_ and D* = D*_Co-DNA_/D*_Control_), which were extracted from the *I*_D_ − *V*_G_ curves of the devices doped by Gd^3+^ and Tb^3+^-based Co-DNA, were then plotted as a function of wavelength in Fig. 8c,d, respectively. As already mentioned in Fig. 7, p-doping of MoS_2_ by Gd^3+^-based Co-DNA and n-doping of WSe_2_ by Tb^3+^-based Co-DNA presented better optoelectronic performance (higher photoresponsivity and detectivity) at 785-nm and 850-nm as compared to the control devices. After doping the MoS_2_ and WSe_2_ devices respectively with Tb^3+^ and Gd^3+^-based Co-DNA, worse performance was observed in the same wavelength range because of the narrowed depletion width. However, under exposure to a 655-nm laser, dramatic performance improvement was observed in the devices doped by Tb^3+^-based Co-DNA, but not in the case of Gd^3+^-based Co-DNA. This seems to be attributed to the excellent emission and absorption properties of Tb^3+^-based Co-DNA around 655-nm, which were confirmed by PL analysis in Fig. 8e. Because of the Tb^3+^ ions attached mainly on the backbone sites, much higher PL intensity was observed in the Co-DNA, where a broad PL spectrum consists of various PL peaks between 438-nm and 630-nm; their corresponding energy state levels are shown in Fig. 8f. Several research groups previously reported the superior emitting properties of Tb^3+^ ions in the range from 380-nm to 700-nm^47^,^48^,^49^. Through the energy state levels of Tb^3+^ ions, the incident 655-nm light is expected to additionally generate electron-hole pairs in the Co-DNA region. After that, we predict that the photocarriers are transferred from the Co-DNA to the TMD layers, consequently increasing the number of total photocarriers and the photocurrent. We also note that Gd^3+^ ions generally show emitting and absorbing properties in the wavelength range between 250-nm and 312-nm, which is far from the wavelengths of the lasers used^49^,^50^,^51^.

## Conclusion

In conclusion, we proposed a new DNA-based doping method on the MoS_2_ and WSe_2_ films (representative n- and p-channel materials, respectively), which enabled ultra-low n- and p-doping control by selecting and/or combining different types of positive ions on DNA nanostructures. Because the PO_4_^−^ in DNA caused n-doping on the TMD films by attracting and holding hole carriers on the side of the TMD, the various selections and/or combinations of the divalent and trivalent positive ions on DNA made it possible to achieve both very low-level n- and p-doping, which are very close to the intrinsic level. The available n-doping range (∆*n*) on the MoS_2_ by Ln-DNA was between 6 × 10^9 ^cm^−2^ and 2.6 × 10^10 ^cm^−2^, which was even lower than that by pristine DNA (~6.4 × 10^10 ^cm^−2^). The p-doping change (∆*p*) on the WSe_2_ by Ln-DNA was controlled from −1.0 × 10^10 ^cm^−2^ to −2.4 × 10^9 ^cm^−2^. These changes were consistent with the red-shift phenomenon in Raman peaks, which indicated n-doping (an increase of electron carriers in MoS_2_ and a decrease of hole carriers in WSe_2_). In the case of Co-DNA doping, where Eu^3+^ or Gd^3+^ ions were incorporated, a light p-doping phenomenon was observed on the MoS_2_ and WSe_2_ (respectively, negative ∆*n* below −9 × 10^9 ^cm^−2^ and positive ∆*p* above 1.4 × 10^10 ^cm^−2^) because the additionally inserted Cu^2+^ ions probably reduced the strength of negative charges in Ln-DNA. However, a light n-doping phenomenon (positive ∆*n* above 10^10 ^cm^−2^ and negative ∆*p* below −1.1 × 10^10 ^cm^−2^) was interestingly observed in the MoS_2_ and WSe_2_ devices doped by Co-DNA with Tb^3+^ or Er^3+^ ions. Compared to the cases of Eu- or Gd-DNA (Ln-DNA), fewer Cu^2+^ ions are thought to be additionally attached to the Tb- and Er-DNA (Ln-DNA), eventually maintaining a negative polarity of Co-DNA. We also found a significant increase in on-current in the MoS_2_ and WSe_2_ devices (by a factor of ~4), which were respectively doped with Tb^3+^-based Co-DNA (n-doping) and Gd^3+^-based Co-DNA (p-doping), due to the reduction of effective electron and hole barrier heights. In terms of optoelectronic device performance (photoresponsivity and detectivity), n-doping by Tb^3+^ or Er^3+^-based Co-DNA and p-doping by the Eu^3+^ or Gd^3+^-based Co-DNA improved MoS_2_ and WSe_2_ photodetectors, respectively. It is thought that the depletion width at the source/TMD junction is broadened after proper Co-DNA doping, consequently helping in collecting photocarriers and increasing photocurrent. In particular, we also found an interesting effect of Tb^3+^ ions on the TMD photodetectors; the Tb^3+^ ions in the Co-DNA region absorbed incident 655-nm light well, generated electron-hole pairs, transferred the carriers to the TMD layers, and finally increased the number of photocarriers and the photocurrents. Ultra-low doping research using amalgamative materials is expected to play a significant role in the improvement of TMD device performance in the short term, and in the continued development of the bio-electronic research field in the long term.

## Experimetal Methods

### DNA DX lattice fabrication

High-performance liquid chromatography (HPLC)-purified synthetic oligonucleotides of DNA was purchased from BIONEER (www.bioneer.com). Two DX tiles were used to construct a 2D DNA nanostructure through a conventional free solution annealing process. Complexes for the DX structure (200 nM) were formed by mixing a stoichiometric quantity of each strand in physiological 1 × TAE/Mg^2+^ buffer (40 mM Tris base, 20 mM acetic acid, 1 mM EDTA (pH 8.0), and 12.5 mM magnesium acetate). They were slowly cooled from 95 °C to 25 °C to facilitate hybridization by placing micro-tubes in 2 L of boiled water in a Styrofoam box for at least 24 hours.

### Lanthanide ion coordination on DNA

For the Ln-ion doping, the individual DNA strands in a total volume of 250 μL were mixed with different concentrations (0, 0.5, 1, and 2 mM) of Er(NO_3_)_3_•5H_2_O, Eu(NO_3_)_3_•5H_2_O, Gd(NO_3_)_3_•6H_2_O, and Tb(NO_3_)_3_•6H_2_O (purchased from Sigma-Aldrich). After annealing DX lattices in a test tube, the appropriate amount of Ln-ion solution was added, and the mixture was then incubated at room temperature for 24 hours.

### AFM measurement on DNA and Ln-DNA

AFM measurement was performed in a substrate assisted grown sample. A DNA-covered sample was attached to the metal puck using instant glue. 20 *μ*L of 1 × TAE/Mg^2+^ buffer was added onto the substrate, and 10 *μ*L of 1 × TAE/Mg^2+^ buffer was mounted onto the AFM tip [A NP-S oxide-sharpened silicon nitride tip (Veeco, USA)]. AFM images were obtained by a Digital Instruments Nanoscope III (Veeco, USA).

### Characterizations of Ln-DNA or Co-DNA-doped TMD films

Ln-DNA or Co-DNA-doped TMD samples were investigated and compared with a control sample (undoped TMD) by PL/Raman spectroscopy (Alpha300 M+, WITec). Here, TMD bulk flakes with similar thickness (~38 nm for MoS_2_ and ~33 nm for WSe_2_) were selected through AFM analysis in order to avoid the thickness effect. Raman spectroscopy with an excitation wavelength of 532 nm was used; the laser beam size was approximately 0.7 ~ 0.9 *μ*m, and the instrumental spectral resolution was less than 0.9 cm^−1^. An integration time of 5 seconds and a spectrometer with 1800 grooves/mm was employed for the test.

### Fabrication of Ln-DNA or Co-DNA-doped TMD electronic/optoelectronic devices

For the fabrication of back-gated TMD transistors, source/drain electrode regions were patterned (channel length and width are 5 *μ*m) on TMD/SiO_2_/Si samples by optical lithography, followed by 10-nm-thick Ti (for MoS_2_) or Pt (for WSe_2_) and 50-nm-thick Au deposition in an e-beam evaporator. After fabrication of TMD devices, ~150 μL volume of pristine DNA, Ln-DNA, or Co-DNA sample solution was dropped onto TMD devices in order to fully cover the TMD surface. After drop-casting the sample on TMD, we incubated the samples in a natural drying environment at room temperature for about 10 hours. We performed this same drop-casting and natural drying process for each TMD transistor five times to ensure full coverage of DNA on the TMD flakes. After finishing this process, TMDs were washed with DI water. Transistors were doped by Ln-DNA or Co-DNA with different metal ions (Eu^3+^, Gd^3+^, Tb^3+^, Er^3+^, or Cu^2+^). Here, the thickness of the gate oxide (SiO_2_) was 90 nm.

### Electrical characterization of Ln-DNA or Co-DNA-doped TMD electronic devices

The fabricated transistor devices were electrically analyzed using an HP 4415B semiconductor parameter analyzer (*I*_D_ − *V*_D_ and *I*_D_ − *V*_G_). The threshold voltage (*V*_TH_) and carrier concentration (*n*) were calculated from *I*_D_ − *V*_G_ data. All drain currents (*I*_D_) were normalized according to the channel width (*W*). For the calculation, we used the equation *n* (or *p*) = *I*_D_*L*/*qWμV*_D_, where *q* is the electron charge and *L*/*W* is the ratio of length to width of the channel. For comparison with other doping studies performed on different numbers of layers of TMD films, the extracted 2D carrier concentration values were also normalized by the number of TMD layers.

### Optical characterization of Ln-DNA- or Co-DNA-doped

In order to investigate the optoelectronic properties of the fabricated Ln-DNA- or Co-DNA-doped TMD devices, a current-voltage (*I*_D_ − *V*_G_) measurement was performed under both dark and illuminated conditions. The light source was a dot laser with wavelengths of 655 nm, 785 nm, and 850 nm, and an optical power of 0.6 mW. For the characterization and comparison of the TMD optoelectronic devices doped by Ln-DNA or Co-DNA with different metal ions (Eu^3+^, Gd^3+^, Tb^3+^, Er^3+^ or Cu^2+^), photo-responsivity (*R*) and detectivity (D*) were calculated from *I*_D_ − *V*_G_ curves. *R* is *I*_Photo_/*P*_Light_ and D* is (*RA*^1/2^)/(2*eI*_Dark_)^1/2^, where *I*_Photo_ is the generated photo-current, *P*_Light_ is the total incident optical power, *A* is the effective area of the detector, *e* is the absolute value of electron charge (1.6 × 10^−19 ^C), and *I*_Dark_ is the dark current.

### Photoluminescence measurement of pristine and Tb^3+^-doped DNA

The emission spectra of pristine DNA and Tb^3+^-DNA on silica substrate were obtained using a fluorescence spectrophotometer (LS-55, PerkinElmer Instruments, USA) at room temperature. The emission spectrum was measured by exciting the sample at wavelength of 400 nm.

## Additional Information

**How to cite this article**: Kang, D.-H. *et al.* Ultra-low Doping on Two-Dimensional Transition Metal Dichalcogenides using DNA Nanostructure Doped by a Combination of Lanthanide and Metal Ions. *Sci. Rep.* **6**, 20333; doi: 10.1038/srep20333 (2016).

## Supplementary Material

Supplementary Information

## Figures and Tables

**Table 1:** Atomic numbers, ionic radii, and optimum concentrations of various Ln ions.

	Lanthanide ions			
	Eu	Gd	Tb	Er
Atomic number	63	64	65	68
Ionic radius (pm)	108.7	107.8	106.3	103
Optimum concentration (mM)	1	1	1	1
