# Supplementary material for: Ultra-low Doping on Two-Dimensional Transition Metal Dichalcogenides using DNA Nanostructure Doped by a Combination of Lanthanide and Metal Ions
Source: Sci Rep. 2016 Feb 3;6:20333. doi: 10.1038/srep20333 (PMC4738262; doi:10.1038/srep20333)
Supplement: Supplementary Information [file srep20333-s1.doc]

**Supplementary Information**

Ultra-low Doping on Two-Dimensional Transition Metal Dichalcogenides Using DNA Nanostructure Doped by a Combination of Lanthanide and Metal Ions

*Dong-Ho Kang^1,+^, Sreekantha Reddy Dugasani^2,3,+^, Hyung-Youl Park^1^, Jaewoo Shim^1^, Bramaramba Gnapareddy^3^, Jaeho Jeon^3^, Sungjoo Lee^1,3^, Yonghan Roh^1,3^, Sung Ha Park^2,3,*^, and Jin-Hong Park^1,*^*

^1^ School of Electronics and Electrical Engineering, Sungkyunkwan University, Suwon 440-746, Korea

^2^ Department of Physics, Sungkyunkwan University, Suwon 440-746, Korea

^3^ SKKU Advanced Institute of Nanotechnology (SAINT), Sungkyunkwan University, Suwon 440-746, Korea

^+^ Equally contributed as the first author.

*Corresponding Authors’ Email Addresses: [jhpark9@skku.edu](mailto:jhpark9@skku.edu) (Jin-Hong Park) and [sunghapark@skku.edu](mailto:sunghapark@skku.edu) (Sung Ha Park).

**Schematic diagram, sequence pool, and sticky-ends for the double crossover (DX) tiles**


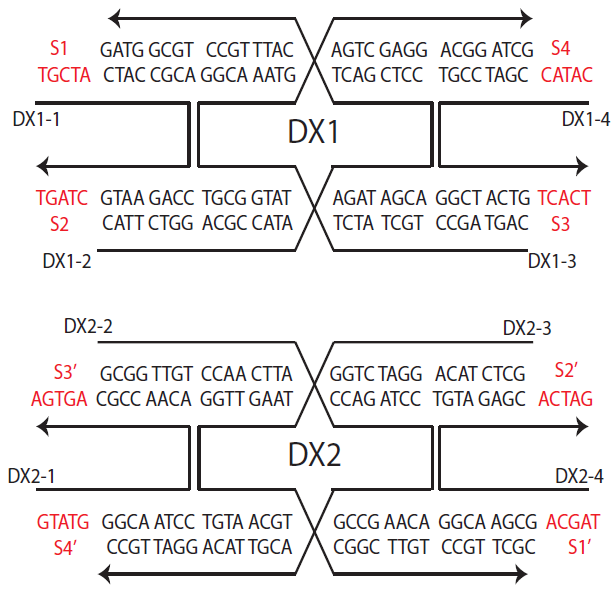


**Figure S1.** A schematic diagram of the double crossover (DX) [DX = (DX1) + (DX2)] tiles. Each tile consisted of four strands: DX1-1, DX1-2, DX1-3, and DX1-4 for (DX1) tiles and DX2-1, DX2-2, DX2-3, and DX2-4 for (DX2) tiles. Complementary sticky end pairs are shown as Sn and Sn’ in the sequence drawings (red).

| **Strand** | **Total # of NTs** | **Sequence (5’ to 3’)** |
| --- | --- | --- |
| DX1-1 | 26 | TGCTA CTACCGCA CCAGAATG CTAGT |
| DX1-2 | 48 | CATTCTGG ACGCCATA AGATAGCA CCTCGACT CATTTGCC TGCGGTAG |
| DX1-3 | 48 | CAGTAGCC TGCTATCT TATGGCGT GGCAAATG AGTCGAGG ACGGATCG |
| DX1-4 | 26 | CATAC CGATCCGT GGCTACTG TCACT |
| DX2-1 | 26 | GTATG GGCAATCC ACAACCGC AGTGA |
| DX2-2 | 48 | GCGGTTGT CCAACTTA CCAGATCC ACAAGCCG ACGTTACA GGATTGCC |
| DX2-3 | 48 | GCTCTACA GGATCTGG TAAGTTGG TGTAACGT CGGCTTGT CCGTTCGC |
| DX2-4 | 26 | TAGCA GCGAACGG TGTAGAGC ACTAG |

**Table S1.** Sequence pool for the double crossover (DX) tiles.

|  | 5’ to 3’ | 3’ to 5’ |  |
| --- | --- | --- | --- |
| S1 | TGCTA | ACGAT | S1’ |
| S2 | CTAGT | GATCA | S2’ |
| S3 | TCACT | AGTGA | S3’ |
| S4 | CATAC | GTATG | S4’ |

**Table S2.** Sticky-ends of the double crossover (DX) tiles shown in Figure S1.

**AFM analysis of the MoS_2_ and WSe_2_ flakes used in this experiment**


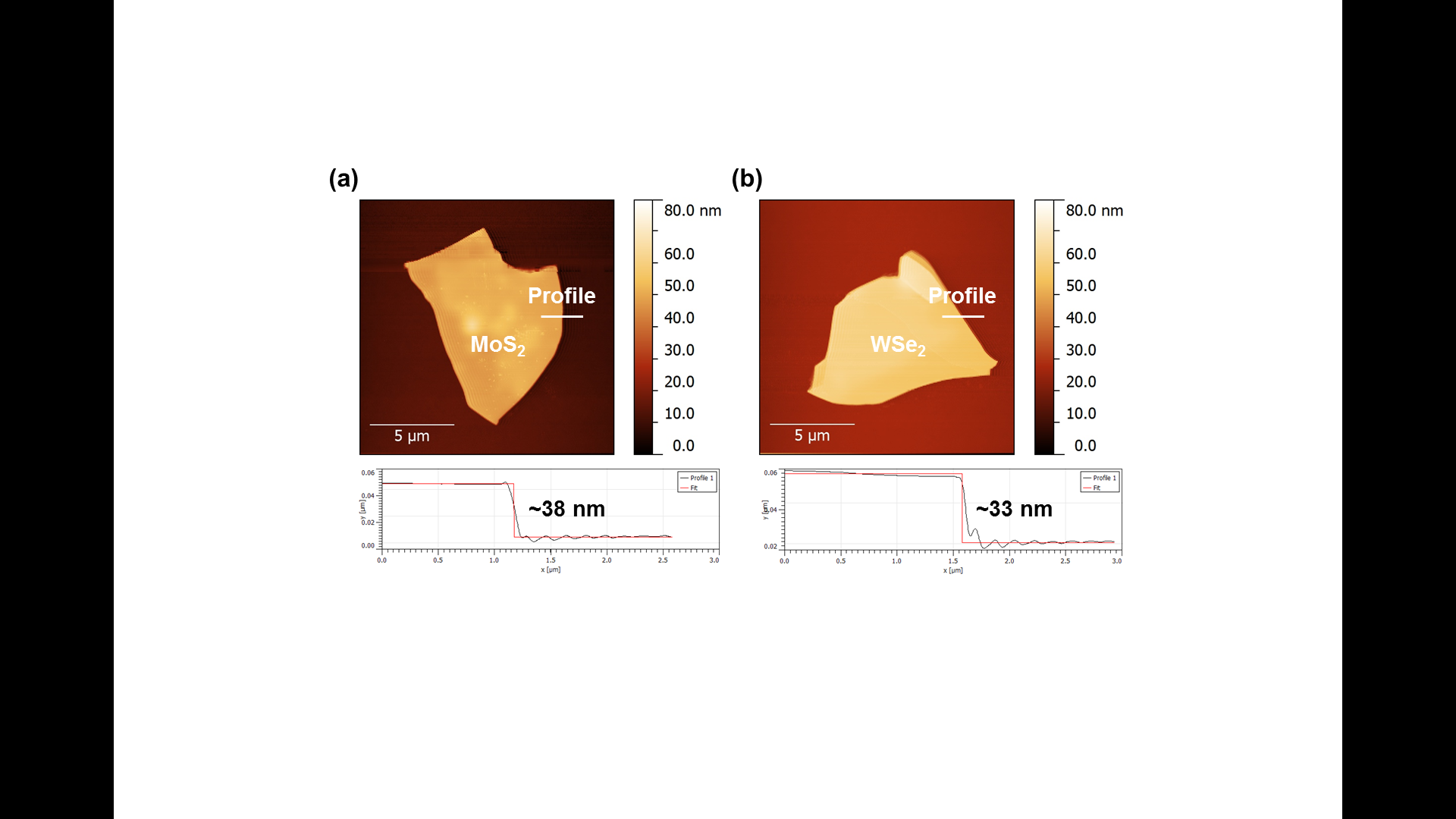


**Figure S2.** AFM images and height profiles for (a) MoS_2_ and (b) WSe_2_ flakes exfoliated on SiO_2_/Si substrates. In this experiment, we selected TMD flakes with a similar thickness (MoS_2_: ~38 nm or 54 layers, WSe_2_: ~33 nm or 47 layers) to avoid the thickness effect.

**Raman spectrum of TMD films doped by Ln- or Co-DNA**


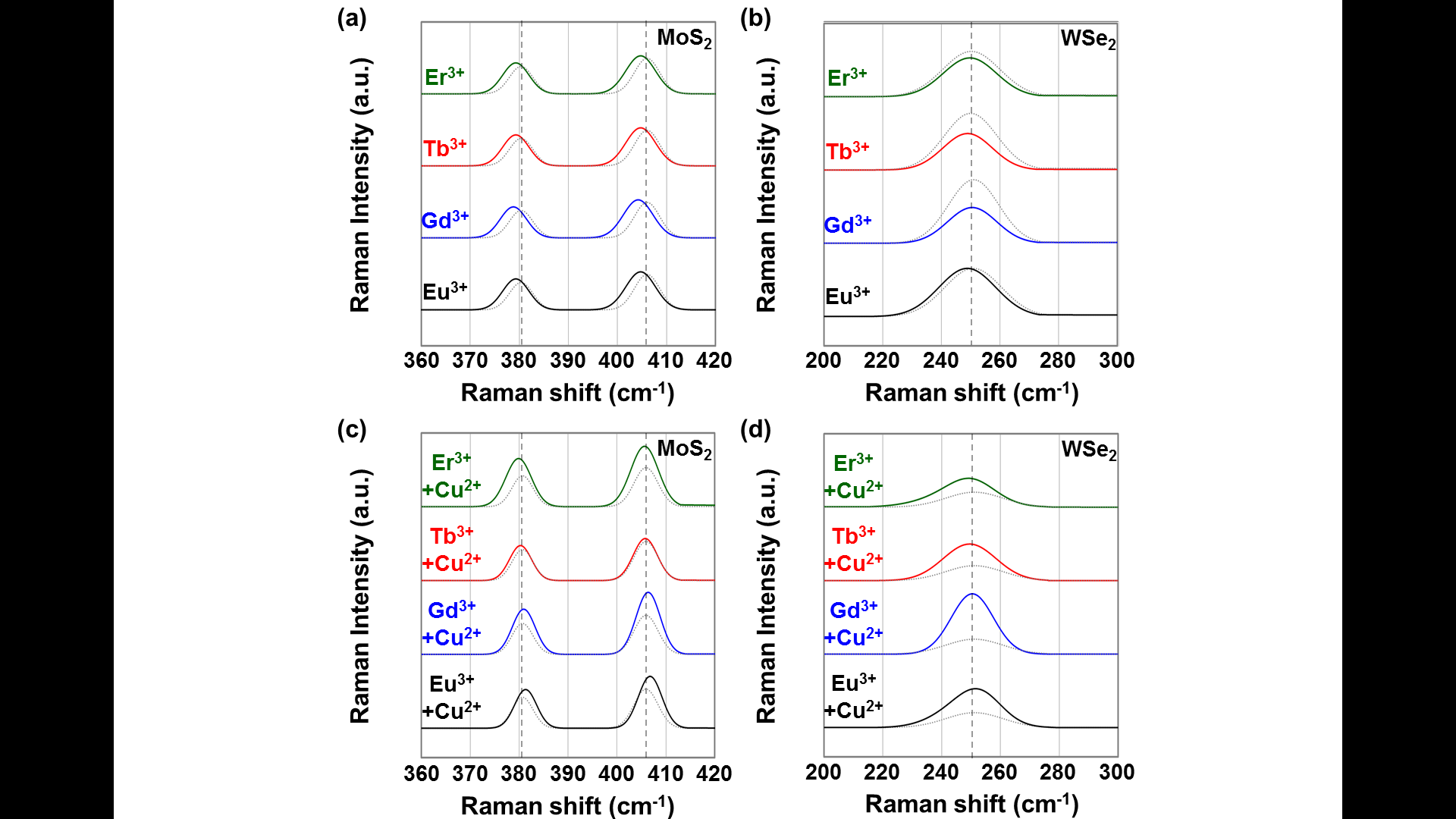


**Figure S3.** Raman spectra measured on (a) MoS_2_ and (b) WSe_2_ doped by Ln-DNA, and (c) MoS_2_ and (d) WSe_2_ doped by Co-DNA.

**AFM images of DNA DX structures where various lanthanide ions are added**


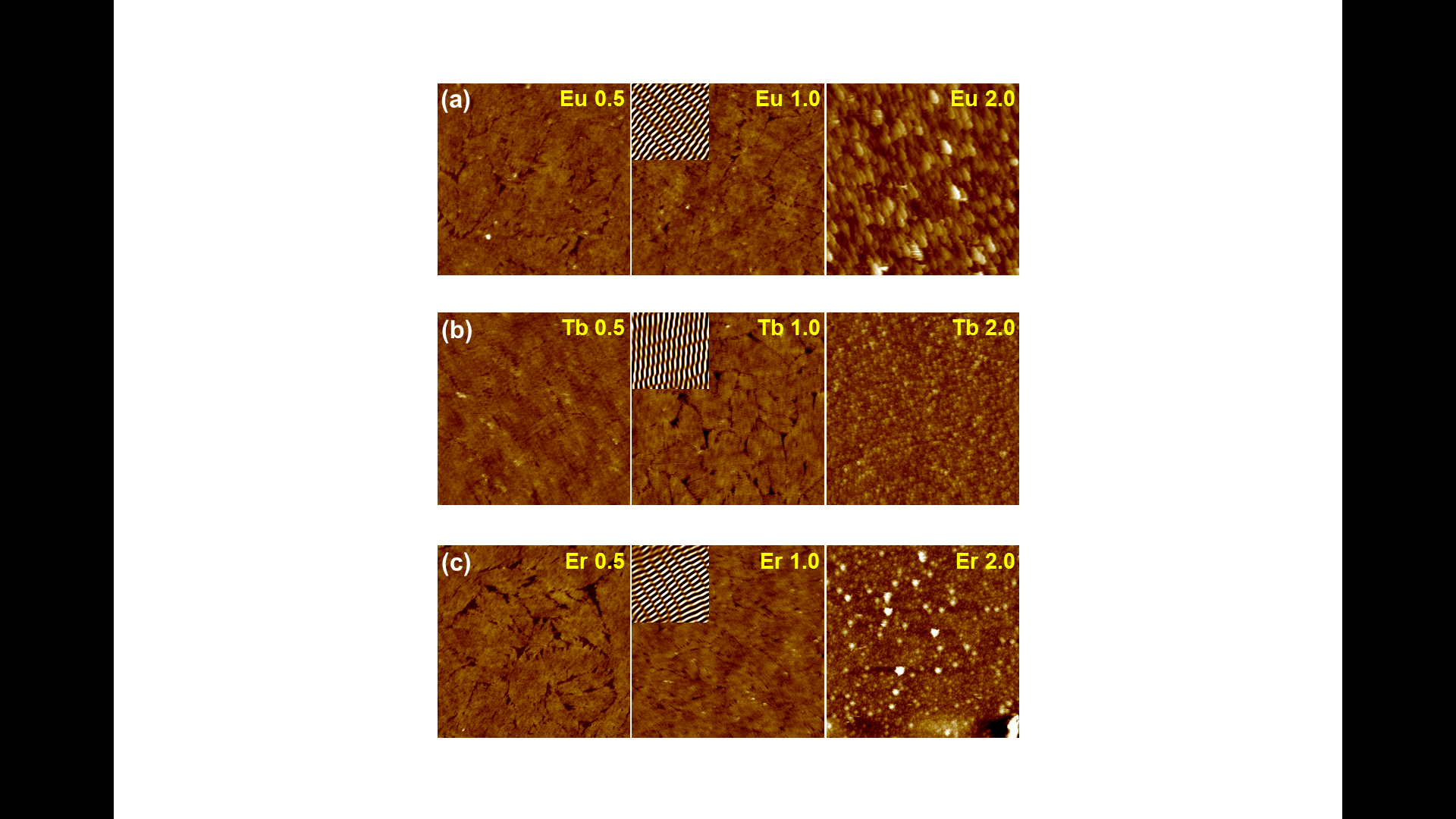


**Figure S4** AFM images of Ln-DNA DX structures where various concentrations of Ln^3+^ ions, such as (**a**) 0.5, 1.0, and 2.0 mM of Eu^3+^, (**b**) 0.5, 1.0, and 2.0 mM of Tb^3+^, and (**c**) 0.5, 1.0, and 2.0 mM of Er^3+^. Here, we observed the structure deformation of Eu^3+^ 2.0 mM, Tb^3+^ 2.0 mM and Er^3+^ 2.0 mM samples, but not in 1.0 mM of all Ln-DNA samples.

**AFM images of Co-DNA DX structures where various lanthanide ions and 2.0 mM of Cu^2+^ ions are added**


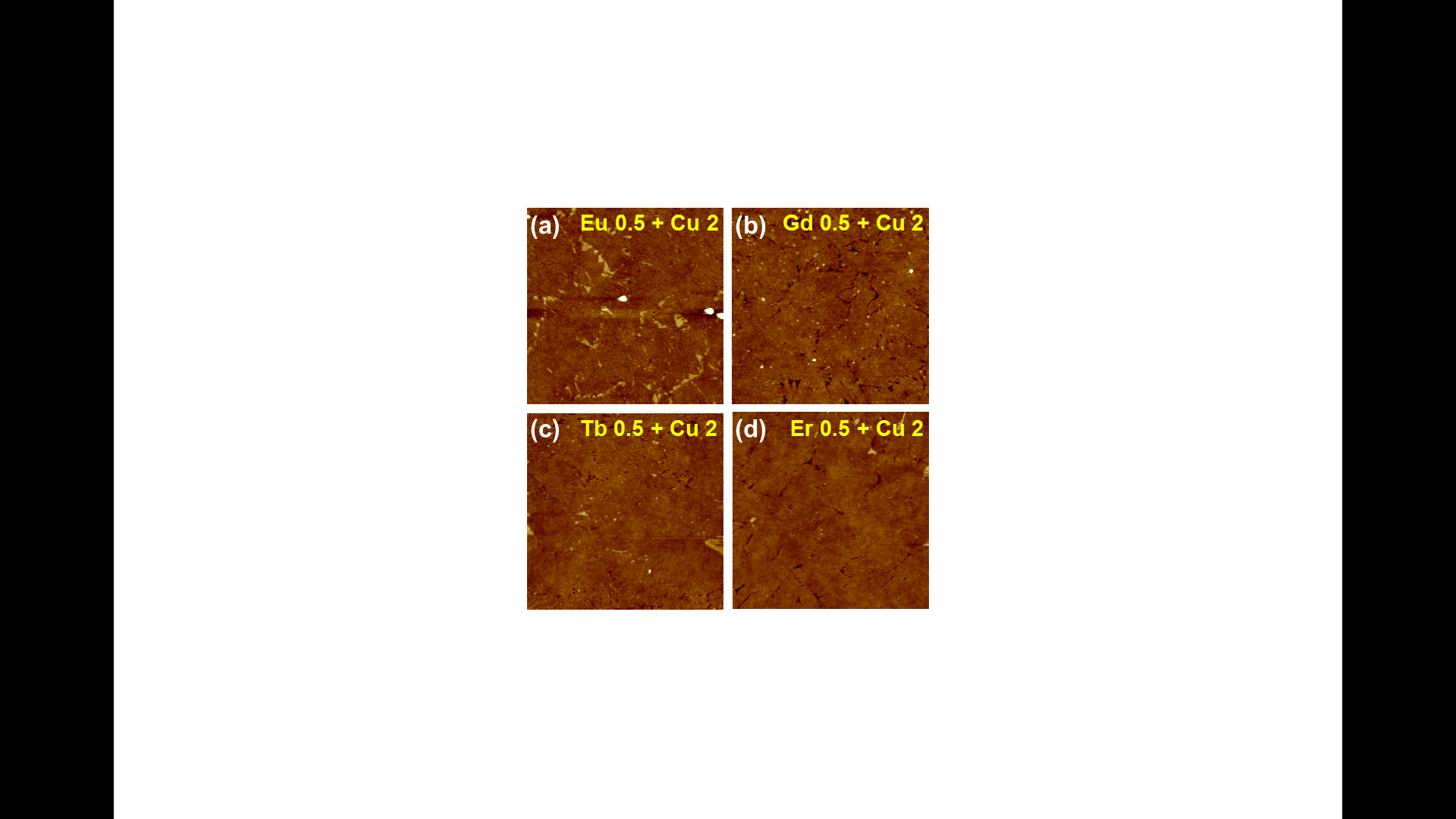


**Figure S6** AFM images of Co-DNA DX structures where various concentrations of Ln^3+^ ions, such as (**a**) 0.5 mM of Eu^3+^, (**b**) 0.5 mM of Gd^3+^, (**c**) 0.5 mM of Tb^3+^, and (**d**) 0.5 mM of Er^3+^. All samples included 2 mM of Cu^2+^ ion equally. Here, we observed the clear Co-DNA structure formations in all samples.

**AFM image and thickness profile of pristine DNA nanostructure under buffer condition.**


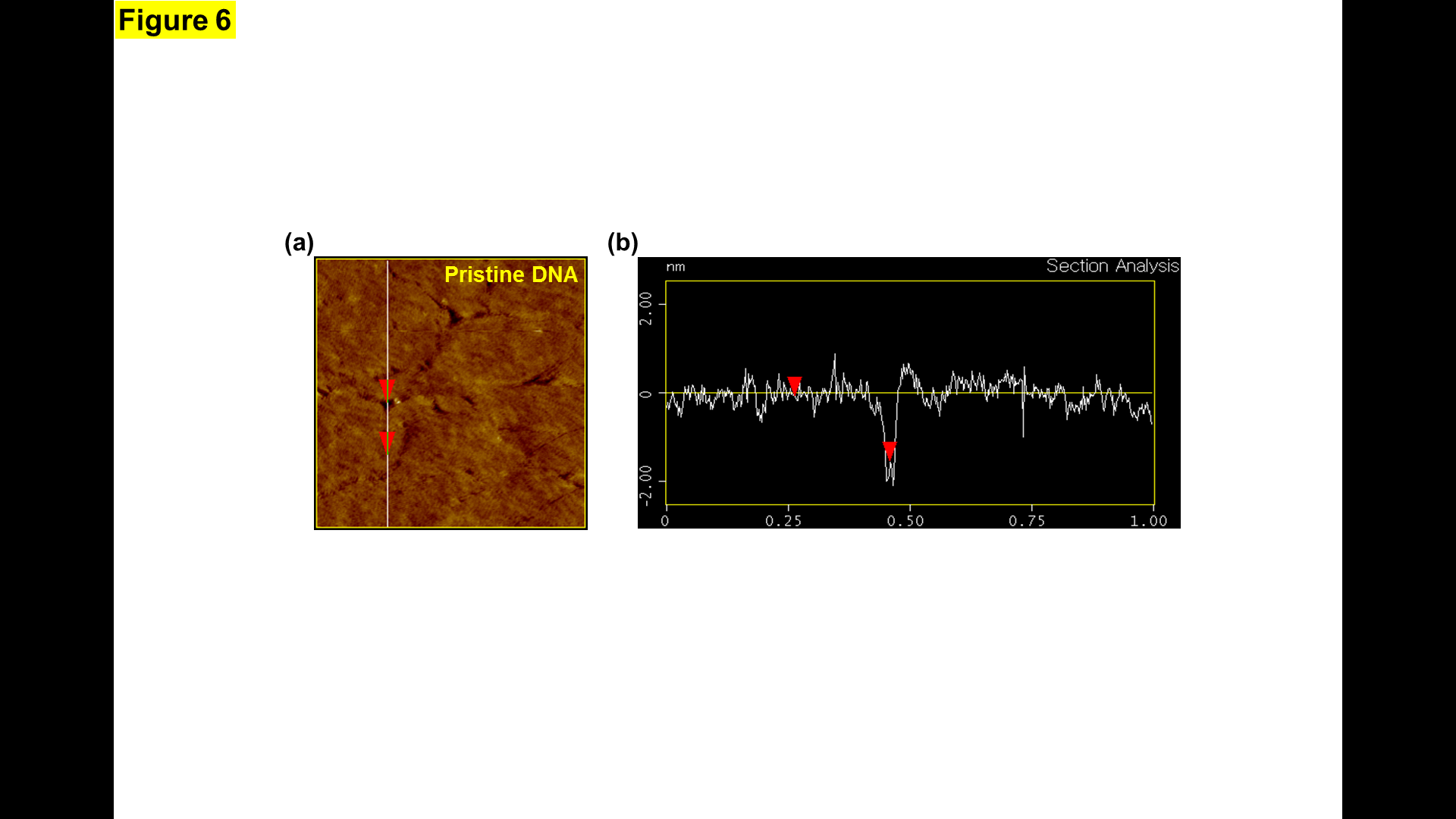


**Figure S6** (a) AFM image and (b) thickness profile of pristine DNA nanostructure under buffer condition.

The thickness of the pristine DNA nanostructure was about 1.5 nm, which is in good agreement with previously reported data.^[ref1]^ It is known that a single-layered DNA is 0.4–0.8 nm in dry conditions and 1.0–1.4 nm under buffer (although the known diameter of the DNA duplex is ~ 2 nm) because of interaction between the DNA and the substrate.

^[ref1]^ S. H. Park et al, *Appl. Phys. Lett*. 2006, *89*, 033901-3

**Threshold voltage monitoring on Ln- or Co-DNA-doped MoS_2_ and WSe_2_ transistors in air.**


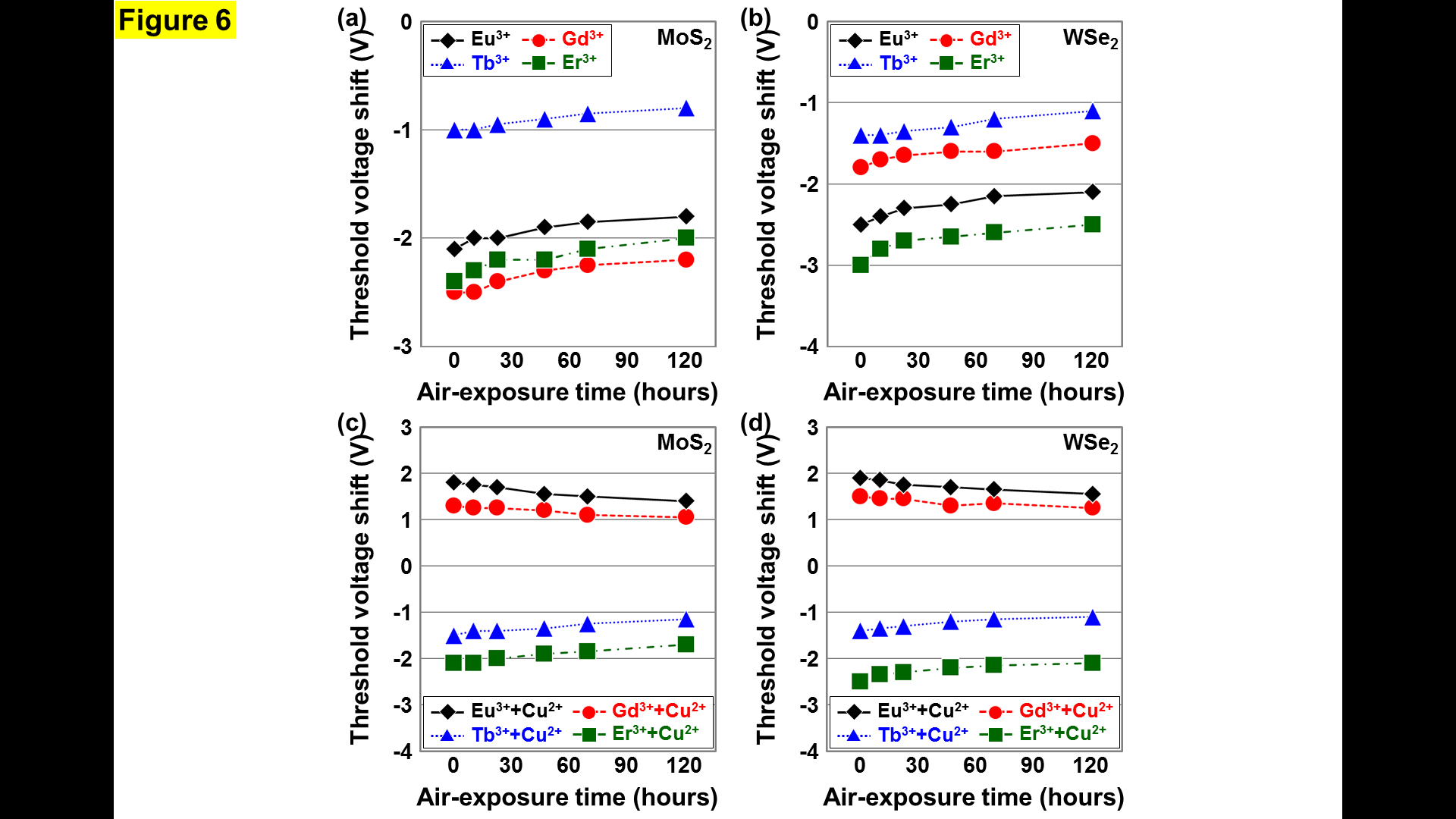


**Figure S7** Threshold voltage shifts of Ln- (a, b) or Co-DNA-doped (c, d) MoS_2_ and WSe_2_ transistors as a function of air-exposure time.

**Ln- and Co-DNA doping effects on TMD devices fabricated on SAM-treated SiO_2_/Si substrate.**


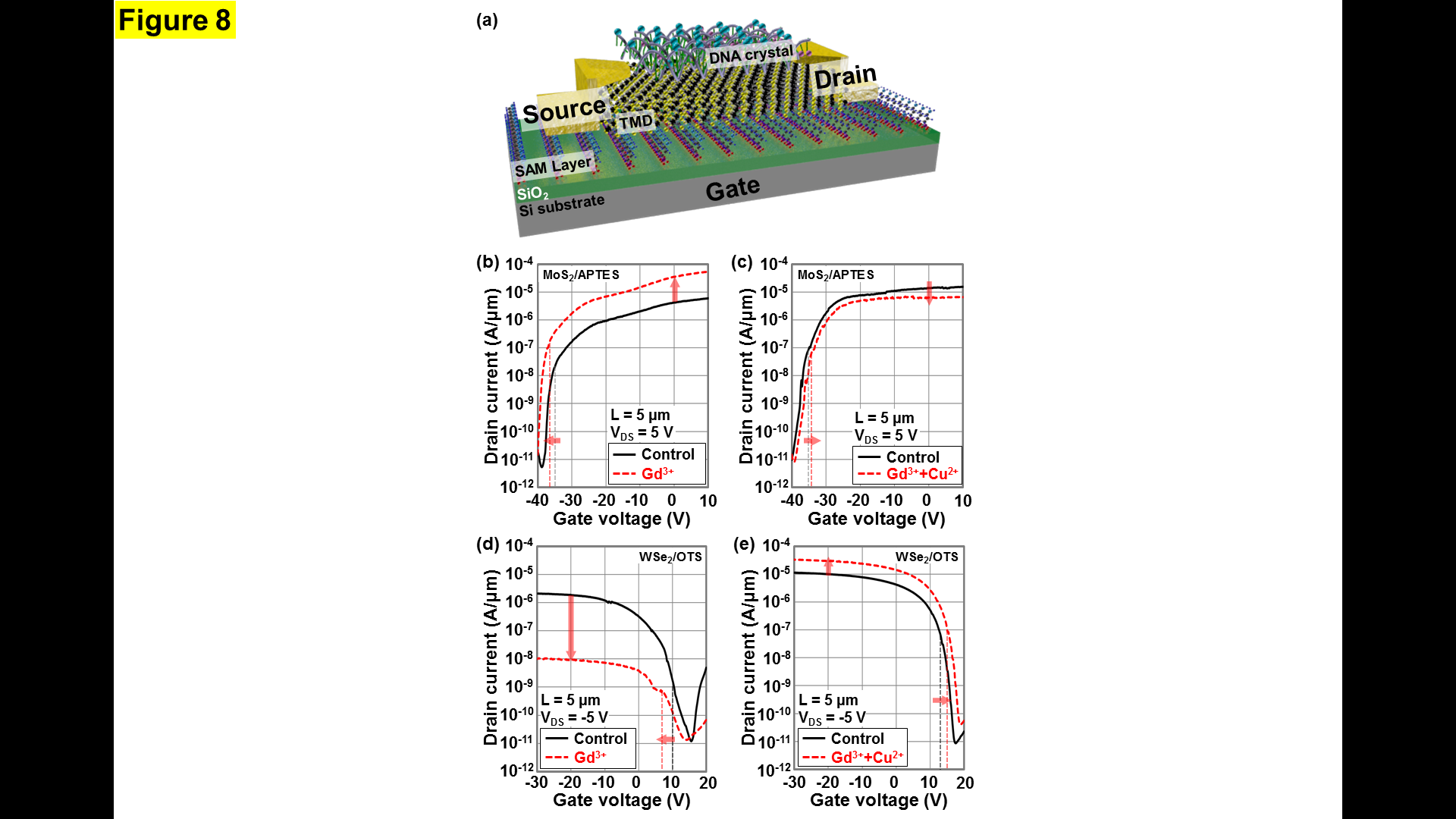


**Figure S8** (a) Schematic illustration of DNA-doped TMD transistors fabricated on SAM-treated SiO_2_/Si substrate. Here, we selected APTES under MoS_2_ and OTS under WSe_2_. I_D_-V_GS_ characteristics of MoS_2_/APTES (b, c) and WSe_2_/OTS (d, e) transistors doped by Gd-DNA (left: b, d) and Gd^3+^-based Co-DNA (right: c, e).

We added the self-assembled monolayer (SAM) molecules between the TMD and SiO_2_ and again performed Ln-DNA and Co-DNA doping on the TMD-based devices to prevent the effects of charge impurities that are not uniformly distributed on the SiO_2_ surface. In this experiment, we used two kinds of SAM layers: APTES (with a negative charge) and OTS (with a positive charge) under MoS_2_ and WSe_2_, respectively. In the case of a Gd-DNA-doped MoS_2_/APTES transistor, the on-current improved by a factor of ~9 (6.0×10^-6^ → 5.4×10^-5^ A/*μ*m), and the threshold voltage shift was ~2.38 V (-34.8 V → -37.2 V). Conversely, in the case of Gd^3+^-based Co-DNA-doped MoS_2_/APTES transistor, the on-current decreased by a factor of 0.4 (from 1.55×10^-5^ to 6.57×10^-6^ A/*μ*m), and the threshold voltage shifted ~1.5 V (from -35.2V to -33.7 V). In the WSe_2_/OTS transistor doped by Gd-DNA, we observed on-current deterioration and a negative-shift in threshold voltage (I_on_ ratio: 0.04 and ΔV_TH_: -2.73 V). The opposite performance changes (on-current improvement and positive-shift in threshold voltage) were confirmed in the Gd^3+^-based Co-DNA-doped WSe_2_/OTS transistor (I_on_ ratio: ~3 and ΔV_TH_: -1.32 V).
